# *Camellia sinensis* in the Prevention and Treatment of Dry Mouth: A Review

**DOI:** 10.3390/dj14060363

**Published:** 2026-06-11

**Authors:** Margaret Conde, Elizabeth Kao, Olivia Schmieder, Macie Watkins, Rachel G. Newman, Janet C. Tou

**Affiliations:** 1 Conde Family Dental, Buckhannon, WV 26201, USA; maggie.conde@condefemalydental.com; 2 Department of Restorative Dentistry, School of Dentistry, West Virginia University, Morgantown, WV 26506, USA; ekao@hsc.edu; 3 Human Nutrition and Foods, School of Agriculture and Food Systems, West Virginia University, Morgantown, WV 26506, USA; ors00009@mix.wvu.edu (O.S.); mw00102@mix.wvu.edu (M.W.); 4 University of Chicago Medical Center, Chicago, IL 60615, USA; newman.rachel@gmail.com

**Keywords:** tea, hyposalivation, xerostomia, salivary pH, dry mouth, quality of life

## Abstract

**Background/Objective**: Persistent dry mouth, associated with poor oral health and lower quality of life (QoL), affects approximately 20% of adults in the global population. Indicating a potential role in nutrition, *Camellia sinensis* tea leaves contain bioactive compounds that may help prevent and manage dry mouth. This review aimed to evaluate the effects of different tea types on salivary flow rate (SFR), salivary pH, and QoL in healthy and at-risk patients and patients with hyposalivation or xerostomia. **Methods**: A systematic review without meta-analysis (SWiM) was conducted. Pre-defined inclusion and exclusion criteria were applied to databases: PubMed, Scopus, Web of Science, and the Cochrane Library. **Results**: Eighteen studies met the eligibility criteria for inclusion in the review. Over 50% of studies investigated either black or green tea, with most conducted in healthy young adults (67%) and predominantly among females. Fifteen of the studies reported that tea intervention improved at least one outcome of interest. In general, green tea improved SFR and salivary pH more consistently than black, oolong, or matcha tea, particularly in at-risk populations and patients diagnosed with xerostomia. **Conclusions**: Tea consumption, particularly of green and black tea, showed a transient enhancement of salivary flow, pH, and QoL, offering a low-cost non-pharmacological approach to supporting oral health. Definitive recommendations were limited by heterogeneity in study interventions and outcome measurements, small sample sizes, and incomplete reporting of study details. However, tea’s potential as an adjunct for the prevention and management of dry mouth warrants further study in larger, well-designed studies that employ standardized protocols.

## 1. Introduction

The importance of nutrition in the management of oral health and in prevention is increasingly recognized in clinical dentistry. Tea made from the leaves of *Camellia sinensis* is the most consumed drink in the world after water, due in part to suggestions of various health benefits [1]. A cross-sectional analysis examining 2725 Chinese men and 3662 women aged ≥65 years found that drinking green tea was associated with reduced tooth loss in men, while black tea was protective in women [2]. The bioactive compounds in green tea, particularly epigallocatechin-3-gallate (EGCG), have antioxidant, anti-inflammatory, and antibacterial effects, with potential use as an adjunctive therapy for preventing and managing dental complications, including dental caries, periodontal disease, and oral cancer [3]. Tea may also play a role in the prevention and management of dry mouth by supporting salivary function.

Dry mouth is characterized by hyposalivation and xerostomia, the objective and subjective measurements of salivary dysfunction, respectively [4]. Studies estimate that dry mouth affects ~22% of the global population. Although xerostomia can occur across all age groups, it is more prevalent in older adults and women, particularly postmenopausal women [5]. Xerostomia results from both systemic and local factors. Local and lifestyle-related factors include: polypharmacy, head and neck radiation, tobacco use, alcohol consumption, and dehydration [6]. Systemic causes include: endocrine disorders (e.g., diabetes mellitus, thyroid disease), autoimmune conditions (e.g., Sjögren’s syndrome, systemic lupus erythematosus, rheumatoid arthritis), and infections such as actinomycosis and hepatitis C [7]. Interestingly, 45.9% of patients with confirmed SARS-CoV-2 infection reported experiencing xerostomia for the first time [8].

Persistent dry mouth increases the risk of poor oral health, including dental caries, periodontal disease, halitosis, candidiasis, and burning mouth syndrome. Clinical effects include: dysphasia, dysgeusia, and difficulty in speaking, chewing and swallowing, which impair quality of life (QoL) [9]. Reduced salivary flow contributes to poor oral health by decreasing mechanical cleansing, salivary acidity, and acid buffering [10]. Cholinergic agents (e.g., pilocarpine, cevimeline) can stimulate residual salivary function, but their use is limited by adverse effects, including cardiovascular complications and exacerbation of gastric ulcers and asthma [11].

A review of *in vitro* studies and animal models of Sjögren’s syndrome found that green tea EGCG protected the salivary glands via multiple molecular pathways. EGCG upregulated aquaporin-5 expression (AQP5), a water channel protein critical for saliva secretion, and SNARE-mediated exocytosis, which is needed for the translocation of AQP5 to the apical membrane. EGCG also preserved actin cytoskeletal organization, which is necessary for coordinated vesicle trafficking and AQP5 localization, and maintained tight junction integrity to stabilize AQP5 at the apical membrane [12]. However, those studies investigated isolated and purified bioactive compounds extracted from tea.

**Figure 1:**
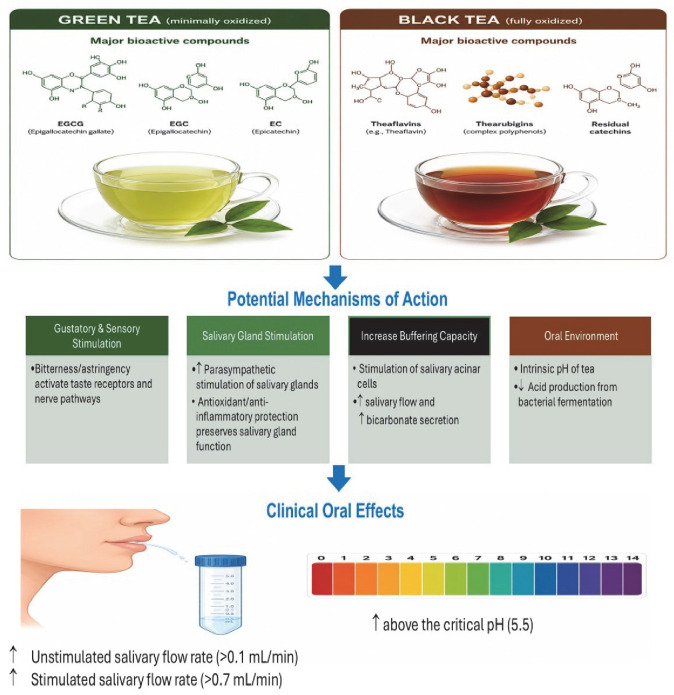
Key bioactive compounds in *Camellia sinensis* and potential mechanisms of action influencing salivary secretion and pH.

Whole tea provides multiple compounds with potential synergistic interactions. The chemical analysis of tea leaves found ~4000 bioactive compounds, with polyphenols contributing to one-third of these compounds [13]. The bitter taste of catechins and the astringency of tannins can activate taste receptors, promoting reflex salivary secretion [14]. Furthermore, the intrinsic pH of tea may transiently shift oral pH away from the critical 5.5 threshold when enamel begins to demineralize [15]. Figure 1 summarizes the potential mechanisms by which the bioactive compounds in *Camellia sinensis* may influence salivary secretion and pH.

Tea’s intrinsic pH and bioactive compounds vary depending on the processing method. Fresh tea rich in catechins forms theaflavins and thearubigins during the oxidation step of tea processing, which can have different effects on salivation and salivary pH [16]. The degree of oxidation determines the type of tea. Black tea is fully oxidized, oolong tea is partially oxidized, and green tea is minimally oxidized [17]. Matcha tea is prepared using whole green tea leaves ground into a fine powder and consumed directly, rather than by steeping leaves and drinking the water extract. As a result, matcha tea has more catechins and a different flavor than green tea [18].

This review evaluated studies investigating the effects of *Camellia sinensis* teas on SFR, salivary pH, and/or QoL in healthy and at-risk patients and patients diagnosed with xerostomia and/or hyposalivation. Inclusion of a diverse population can inform both preventive and therapeutic strategies for dry mouth. As one of the most widely consumed beverages worldwide, tea has the potential to offer a familiar, convenient, low-cost, and safe method to manage dry mouth.

## 2. Methods

This review employed a systematic search and study selection approach guided by the Synthesis Without Meta-analysis (SWiM) framework [19] (Supplementary Table S1). Studies on *Camellia sinensis* teas and dry mouth were highly heterogeneous in terms of populations, tea types, interventions, and outcomes. The use of a systematic search and study selection process provided transparency and reproducibility while retaining flexibility to enable a critical appraisal of the evidence and identification of gaps in the literature.

### 2.1. Eligibility Criteria

Criteria were developed using the Participants, Intervention, Comparator, Outcomes, and Study design (PICOS) framework (Table 1). Only human intervention trials were included. Population criteria included: healthy females and males aged ≥17 years with xerostomia and/or hyposalivation, at risk of dry mouth, or healthy. Eligible interventions were black, oolong, green, or matcha tea provided as a beverage, lozenge, or mouth rinse. Comparators included teas, beverages, or mouth rinses not made from *Camellia sinensis*, placebo, and no intervention (pre- and post-study design). Eligible studies measured at least one of the outcomes of interest: SFR, pH, and/or QoL. Other inclusion criteria were original, peer-reviewed studies written in English.

Exclusion criteria for the population were individuals aged <17 years due to their potential sensitivity to caffeine and other bioactive compounds found in tea and challenges with saliva collection in young children [20]. Ineligible interventions were teas not derived from *Camellia sinensis* (e.g., herbal tea or infusions), studies that did not report the tea type or those that used purified isolated tea extracts (e.g., catechins, EGCG, etc.). Exclusion criteria for study design included animal studies and *in vitro* studies, as reviews already exist. Human observational study design (e.g., cross-sectional, cohort, case-control) and case reports or case series were excluded.

### 2.2. Search Strategy

In consultation with a librarian, a comprehensive search strategy was developed using a combination of pre-tested search terms. Controlled vocabulary terms included medical subject heading terms as well as free-text terms that included phrase searching. A search was conducted in the electronic databases PubMed, Scopus, Web of Science, and Cochrane Library from database inception to February 2025. Appendix A shows the full search strategies for each database. A hand search was conducted in Google Scholar. Studies identified by the search were uploaded to the reference manager Zotero (George Mason University, Fairfax, VA, USA) for de-duplication and then imported into the systematic literature review software manager, Rayyan (a free app https://rayyan.ai/users/sign_in, accessed on 26 May 2026) (Rayyan Systems Inc., Cambridge, MA, USA), to conduct screenings and data extraction.

### 2.3. Screening of Studies and Data Extraction

The studies underwent two screenings using a pre-tested screening form (Appendix B). The initial screening reviewed titles and abstracts for eligibility. Articles rated as “yes” or “maybe” were advanced to a full-text assessment for eligibility. Both screenings were conducted independently by two reviewers, OS and MW. Conflicts were resolved by discussion between reviewers and consultation with a third reviewer, JCT. Data extraction was conducted independently by reviewers OS, MW, and JCT using a pre-tested form (Appendix C). The screening and extraction forms were pre-tested on 15 studies, with a threshold for validation set at 80% inter-rater agreement among reviewers.

### 2.4. Critical Appraisal

Risk of bias was independently assessed by reviewers JCT and RGN, with disagreements resolved through discussion. Due to the heterogeneity of included study designs, methodological quality was appraised using the Mixed Methods Appraisal Tool (MMAT), which enables structured evaluation of randomized and non-randomized studies [21]. Studies were assessed against five design-specific criteria and rated as “Yes,” “No,” or “Can’t tell.” Studies with multiple “No” or “Can’t tell” ratings were considered to have higher risk of bias. Results were entered into an Excel template to generate traffic light plots.

## 3. Results

**Figure 2:**
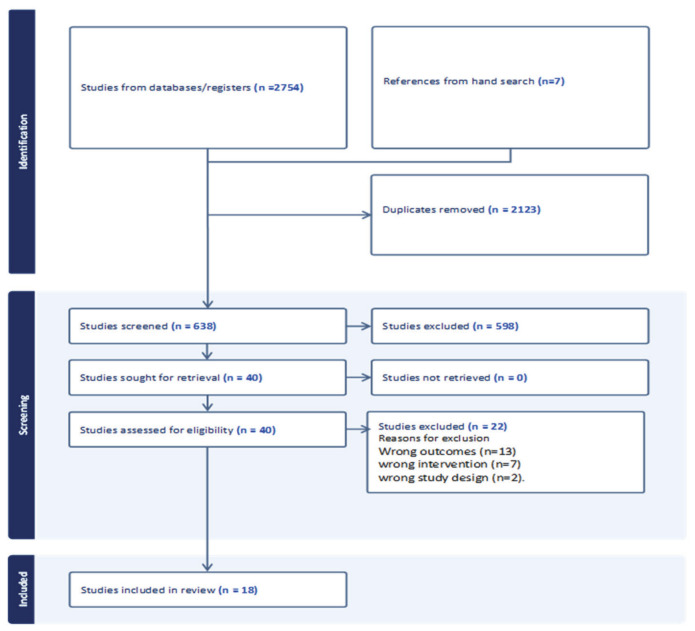
PRISMA Flowchart.

The PRISMA flowchart (Figure 2) summarizes the articles identified and screened. A total of 2754 citations were identified, and seven were identified by hand search. De-duplication of studies resulted in 638 citations for the screening of titles and abstracts for eligibility. A total of 598 studies were excluded in the initial screening, which resulted in a total of 40 studies that were eligible for full-text screening. Following full-text screening, 22 studies were excluded for the following reasons: wrong outcome (n = 13), intervention (n = 7), and study design (n = 2). A total of 18 studies met the eligibility criteria and were included in this review. The eligible studies were grouped thematically by outcomes, i.e., SFR, salivary pH, both, and QoL (Table 2).

## 4. Discussion

### 4.1. Salivary Flow Rate

Hyposalivation is diagnosed by sialometry, a non-invasive 5–15 min test that assesses salivary gland function by measuring SFR. Normal SFR is ~0.4–0.5 mL/min [40]. Hyposalivation is diagnosed when unstimulated SFR (USFR) is ≤0.1 mL/min or stimulated salivary flow rate (SSFR) is ≤0.7 mL/min. Of the five eligible studies examining tea intervention on SFR, three studies involved healthy participants. All studies provided tea as a beverage and measured USFR post-consumption. Three studies investigated a single tea type, while two studies compared tea types (Table 2).

In healthy participants, in a single-arm study comparing tea types, a total of 12 adults (50% male and 50% female) drank oolong and Lapsang souchong black tea. Both tea solutions were prepared by steeping loose tea leaves (1 g) in 30 mL of boiling water, twice, which is a relatively high concentration compared to typical tea preparations. Subsequent chemical analysis of teas found higher phenolics and flavonoids in black than in oolong tea. USFR measured by the passive drooling method showed that neither tea affected USFR at any of the time intervals after consumption [22]. A separate follow-up study analyzing the individual responses of the 12 participants drinking Lapsang souchong tea found high inter-individual variability, with USFR ranging from 0.15 to 0.54 mL/min at 3 min and from 0.10 to 0.52 mL/min at 30 min [23], which may have contributed to the lack of statistical significance.

In both studies, black tea was consumed at 50 °C [22,23]. Using black tea as an astringent stimulus at 0 °C was found to be more effective at increasing salivary flow than at 70 or 37 °C [41]. Therefore, cold liquid stimuli may be a better salivary stimulant in clinical applications than warm or hot liquids. However, a study of healthy adults reported no significant differences in drinking hot (57 ± 0.5 °C) versus cold (8 ± 0.5 °C) black tea [24] on salivary flow. These findings suggest limited effects of black tea on salivary flow in individuals with normal salivary function.

In at-risk participants, Fitri et al. [25] compared the effect of black tea consumption in adults with or without caries because hyposalivation has been associated with a higher caries risk [42]. Participants were assigned (n = 13/group) to drink 1.33% black tea prepared by adding 2 g of tea to 150mL of boiling water, then cooling to 50 °C. USFR was measured 30 min after drinking tea by the passive drooling method. Consumption of black tea increased USFR (*p* < 0.05) in both groups, with no significant differences between the caries and caries-free groups. However, at baseline, there was no significant difference in USFR between the caries (0.52 mL/min) and caries-free (0.56 mL/min) groups, with values in both groups within the normal SFR range (~0.4–0.5 mL/min).

In participants diagnosed with dry mouth, tea intervention resulted in a significant increase in USFR. Menopausal (age 45–65 years) women with xerostomia based on USFR <0.2 mL/min were randomly assigned (n = 20/group) to green tea or water (control). The 15% green tea was prepared by adding 3 g of green tea to 20 mL of hot water, cooling for 5 min, and then filtering. In contrast to previously described black tea intervention studies, green tea was gargled, followed by collection using the spitting method. Both actions cause mechanical stimulation, with the potential to increase salivation [43], which limits comparability with previous black tea studies. Baseline USFR was 0.14 mL/min, confirming xerostomia in both groups. Drinking green tea resulted in significantly greater USFR (0.43 ± 0.13 mL/min) than drinking water (0.18 ± 0.04 mL/min) [26].

**Figure 3:**
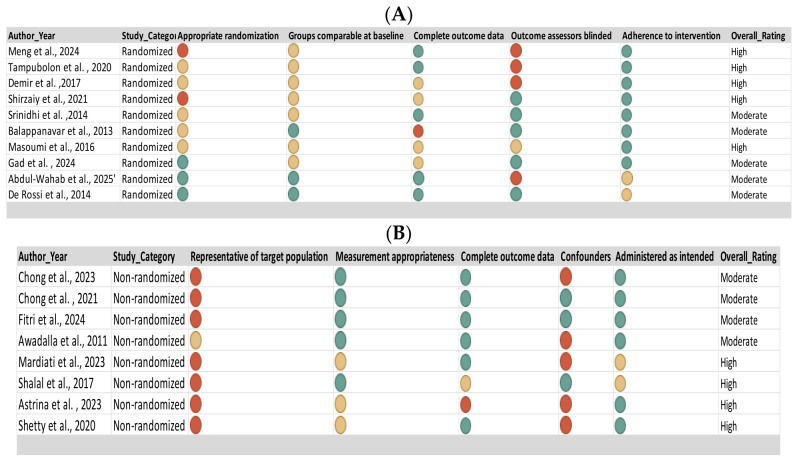
Traffic light plots of risk of bias in included studies assessed using MMAT criteria in (**A**) randomized intervention trials: [24,26,27,28,30,31,36,37,38,39] and (**B**) non-randomized intervention trials: [22,23,25,29,32,33,34,35]. Red indicates high risk of bias, green indicates low risk of bias, and yellow indicates unclear risk of bias.

Interpreted according to participants’ oral health status, findings in healthy individuals were inconsistent, while greater improvements were observed in at-risk individuals and those with xerostomia. Collectively, findings suggest that tea interventions may have greater effects in individuals with impaired salivary function than in those with normal salivary flow. However, the included RCTs were rated as having a high risk of bias, mainly due to a lack of blinding (Figure 3A), while the non-randomized studies were rated as having a moderate risk of bias, mainly due to selection bias (Figure 3B). In Chong et al. [23], baseline USFR among healthy participants ranged from 0.080 to 0.272 mL/min, suggesting that some individuals may have met the criteria for hyposalivation. Similarly, at-risk participants had a baseline USFR within the normal range [26]. This overlap in baseline salivary function may have introduced selection and misclassification bias, limiting the comparability of outcomes between groups.

### 4.2. Salivary pH

A positive correlation exists between SFR and salivary pH. As SFR increases, the concentration of bicarbonate in the saliva increases, which results in a rise in salivary alkalinity [44]. Therefore, salivary pH can serve as another biomarker for evaluating oral hydration. Normal salivary pH ranges from 6.8 to 7.4 [45]. The intrinsic pH of tea can cause a transient change in oral pH. Of the five eligible studies examining tea intervention on salivary pH, four of the studies involved healthy participants and measured salivary pH post-consumption using a pH meter. Three studies provided tea as a beverage, while two of the studies used tea as a mouth rinse (Table 2).

In healthy participants, green tea was compared to the consumption of popular drinks in Turkey. Demir et al. [27] randomly assigned 28 adults to drink either green tea, cola, coffee, or a yogurt beverage. Post-consumption, participants were given paraffin to chew to stimulate salivation for collection at 1, 10, and 30 min. Drinking green tea resulted in a higher salivary pH at 1 min and 30 min compared to the other beverages, including the yogurt (alkaline) beverage. Another study compared the effect of different *Camellia sinensis* teas on salivary pH [28]. Fifty adult males were assigned to drink either black or green tea. Post-tea consumption, saliva was collected by the spitting method at 5 and 10 min. Both teas increased salivary pH, with green tea resulting in a higher (*p* < 0.05) salivary pH than black tea. The double-blind crossover study design minimized variability by allowing each individual to act as their own control. However, potential carryover could not be assessed because the washout duration was not reported. Also, the tea preparation methods differed. The black tea was prepared by adding 2 g loose leaves to 150 mL boiling water and steeping for 3 min, while the green tea was prepared by adding 3 g loose leaves in 150 mL 70 °C water for 3 min. These differences in dose and temperature can alter the chemical composition of tea, limiting the ability to draw definitive conclusions based on tea type. Because green tea is typically brewed at lower temperatures in real-world settings, analyzing and reporting the chemical composition of teas prior to intervention are essential for accurately interpreting the findings.

Two studies investigated tea as a mouth rinse, which prolongs contact with oral tissue compared to drinking tea. Healthy adults rinsed with water and then 1 h later rinsed with 2% green tea for 5 min. Saliva was stimulated by chewing paraffin and collecting at 3, 7, 11, 20, and 30 min. The results showed that rinsing with green tea led to a significant decrease in salivary pH by 0.49 units, but it still remained within the normal pH range of 6.43 ± 0.16 [29]. The authors suggested that the catechins in green tea were responsible for maintaining salivary pH. However, the specific timing of the pH change was not clearly reported, and the pilot study’s lack of control groups for comparison limited the strength of the findings.

In a parallel-designed randomized controlled trial (RCT), healthy adults (10/group) were provided a green tea or chlorhexidine (0.2%) mouth rinse (positive control) [30]. To prepare the 0.5% green tea mouth rinse, 3.5 oz (~103 g) green tea was added to 4 cups (~950 mL) non-sparkling mineral water and steeped for 1 h at room temperature. This green tea stock solution (500 mL) was then diluted in distilled water (1 L). Study participants were instructed to rinse with 15 mL of green tea for 30 s twice daily for 3 weeks, after brushing their teeth. The method used to collect saliva after rinsing was not reported. Salivary pH was measured immediately after the first rinse and at 1, 2, and 3 weeks using pH strips. Both green tea and chlorhexidine showed a rise, although not statistically significant, in saliva pH, with the highest increase (6.44 ± 0.39) occurring at week 3 for the green tea mouth rinse. However, a comparison with chlorhexidine mouth rinse could not be made, as its use was limited to a 2-week duration due to safety considerations.

The effect of different tea types was also investigated in at-risk populations. Caries have been associated with oral acidity [46]. In a double-blind, parallel study, patients with caries or without caries were randomly assigned (10/group; sex differences not reported) to drink either black or green tea [31]. Both teas were prepared by adding one tea bag to 90 mL of boiled water and steeping for 3 min. Saliva was collected at baseline, immediately, and at 5 and 10 min after consumption by aspirating from the sublingual region. Salivary pH increased immediately in both tea types, then gradually decreased but remained higher than at baseline. Green tea had a greater (*p* < 0.05) immediate increase in salivary pH compared to black tea, but the differences were not significant at 5 min. There were no differences in salivary pH between caries and caries-free participants, with both groups’ baseline pH within the normal range. Drinking green and black tea significantly increased salivary pH over time. The rise in salivary pH after green tea was greater in participants with caries, while black tea showed no group differences. These findings suggest that both green and black tea may transiently increase salivary pH in at-risk populations, with green tea demonstrating a greater immediate alkalizing effect.

Collectively, the studies reported that drinking green tea increased salivary alkalinity in both healthy and at-risk populations; however, the evidence for at-risk participants was limited, and findings in healthy participants should primarily be interpreted as mechanistic rather than therapeutic evidence. Tea mouth rinses showed less consistent effects. However, interpretation was limited by methodological heterogeneity. One study used pH strips [30], which are less accurate than pH meters. Also, saliva collection methods can influence salivary pH by affecting salivary flow, which makes it unclear whether the increased pH was due to changes in salivary flow or to the intrinsic pH of the tea. Two studies collected stimulated saliva [28,30], which increases SFR and bicarbonate content and thereby results in a higher pH than USFR. The sublingual aspiration method was used in one study [31]. The sublingual glands contribute little to whole saliva, with the submandibular glands being the primary source of unstimulated saliva and the parotid glands the main source of stimulated saliva [47]. Although all but one study were RCTs, the included studies were rated as having a high to moderate risk of bias, mainly due to concerns regarding unclear randomization (Figure 3A), which may have influenced participant behavior, researcher conduct, and outcome assessment.

### 4.3. SFR and Salivary pH

Given the association between SFR and salivary pH, assessing both biomarkers simultaneously can provide a more comprehensive evaluation of the effects of tea interventions on dry mouth. Of the six eligible studies investigating tea intervention on both SFR and salivary pH, two studies involved participants at risk of dry mouth. Three studies compared tea types, and two studies involved rinsing rather than drinking tea. Two studies measured USFR by the spitting method, and all but one study measured salivary pH using a pH meter (Table 2).

In healthy participants, a quasi-experiment involving 20 women reported that drinking black tea significantly increased SFR and pH [32]. Similarly, in a study of 255 men and women (sex distribution not reported), black tea was consumed after preparation by boiling for 7 min, then cooling for 3 min. USFR was determined at 3 min after consumption by the spitting method, and salivary pH was measured by a pH meter. Black tea increased USFR and salivary pH (*p* ≤ 0.01), with the authors attributing the effects to the activation of taste receptors [33]. A quasi-experimental design can provide valuable insight into causal relationships in real-world settings, provided that steps are taken to minimize potential confounders and biases [48]. However, this study lacked reporting of participant characteristics, tea dose, preparation method, and SFR collection method, which limited the interpretability and reproducibility of the positive findings of drinking black tea [32].

In a study investigating green tea intervention, females were provided tea prepared by adding green tea powder (250 mg) to 100 mL of 80 °C water. After participants gargled and then swallowed the tea, USFR was determined at 5 min by the draining method, and salivary pH was measured by a pH meter. The results found increased SFR and salivary pH (*p* < 0.05) [34]. Green tea is richer in catechins, particularly EGCG, than black tea, and it has been shown to stimulate taste receptors [49]. Comparing tea types, Shetty et al. (2020) [35] assigned (30/group) adults (50% males and 50% females) to rinse with either black or green tea. Both 0.2% teas were prepared by dipping 2 g tea bags in 100 mL warm water (temperature not specified) for 5 min. After participants rinsed with tea for 1 min, SFR and pH were measured at different time intervals. Both tea types increased SFR and salivary pH, with black tea showing a greater (*p* = 0.03) increase in salivary pH and a tendency (*p* = 0.06) for higher SFR than green tea.

Black tea contains fewer catechins than green tea because oxidation converts catechins into theaflavins and thearubigins [50]. Theaflavins have been shown to have greater stability compared to catechins and to act as a mouth-coating agent resulting in long-lasting saliva stimulation [51,52]. However, the chemical composition and pH of the green and black tea preparations were not analyzed to address the underlying mechanisms for the observed differences between tea types. Additionally, the brewing temperature, which can influence the extraction of bioactive compounds, was not specified, and the method used to measure SFR was not reported, which limited the interpretation and reproducibility of the findings.

A study conducted on oral health in Americans found that 40–60% experience periodontitis [53], a population reported to have hyposalivation and acidic pH [54], whereas nearly 90% of adults had dental caries [53]. Patients with dental caries exhibit altered saliva, specifically, reduced SFR, lower salivary pH, and buffering capacity, which impairs the mouth’s ability to neutralize acids that promote the demineralization of enamel resulting in caries [55].

In at-risk participants, men and women (sex distribution not reported) with or without periodontitis [36] were randomly assigned (10/group) to rinse with either black tea or green tea. Both teas were prepared by steeping one tea bag in 250 mL of hot (80–90 °C) water for 2–3 min. After rinsing with tea, USFR was measured by the spitting method, and salivary pH was measured using a pH meter at different time intervals. In the periodontitis group, rinsing with green tea, but not black tea, increased both SFR and pH (*p* ≤ 0.02). The healthy group showed no effect of either tea on SFR or pH. The results suggested that green tea was more beneficial than black tea for patients with periodontitis. This contrasted with the findings in healthy individuals, for whom it was reported that black tea increased SFR compared to green tea [35].

In a double-blind RCT [37], patients at high risk of caries were assigned to drink green tea or matcha tea. Because matcha tea involves consuming the whole leaf rather than steeping and removing leaves, matcha typically contains significantly higher concentrations of catechins, specifically, EGCG, compared to standard brewed green tea [56]. Post-consumption of teas, USFR was measured by passive drooling at 0, 5, and 10 min. The results showed that both tea interventions increased USFR at 5 min and salivary pH at 5 and 10 min. Green tea resulted in a significantly higher USFR but a similar salivary pH compared to matcha tea consumption. Matcha tea’s thicker consistency may have limited its ability to stimulate salivation, while its catechin content produced pH changes comparable to those from green tea consumption. However, no chemical or texture analysis of the teas was conducted. The interpretability and generalizability of the study results were limited by the lack of definition of the threshold used to classify high-caries-risk patients, as well as missing demographic details regarding sex distribution and age, with only the targeted ages of 20–45 years provided.

When interpreted according to participant salivary health status, findings in healthy adults suggested that black tea may have comparable or stronger effects on salivary pH than green tea, possibly due to its intrinsic pH. In at-risk participants, greater improvements in SFR and/or salivary pH were generally observed following green tea consumption; however, the evidence was limited by the small number studies and overall study quality. The four non-randomized studies were rated as having a high risk of bias, mainly selection bias and confounding. The RCTs indicated that at-risk participants consuming green tea showed greater improvements in SFR and/or pH. Tea mouth-rinse interventions also differed by health status, with green tea being more beneficial than black tea in at-risk populations. However, the RCTs were rated as having a high to moderate risk of bias. Additionally, 50% of the studies were missing demographics [33,36,37], which made it difficult to determine whether the outcomes were due to the tea intervention or to participant characteristics. Although all studies measured USFR, saliva collection varied across studies, including the spitting method [33,36], the draining method [34], and passive drooling [37], while two studies did not report the method used to measure SFR [32,35]. These methodological differences may contribute to the mechanical stimulation of salivary secretion, which makes it difficult to definitively conclude which tea type was most effective for increasing both SFR and salivary pH.

Saliva contains a wide variety of biomarkers that can be used to assess physiological and pathophysiological states, and it has the advantages of being non-invasive and allowing serial sampling. Advances in test accuracy, sensitivity and precision for saliva have further improved its diagnostic performance [57]. However, some patients may still report xerostomia with normal SFR, and low oral pH can contribute to the sensation of oral dryness [58]. Therefore, assessing QoL provides critical information on how oral health affects overall well-being.

### 4.4. Quality of Life

Of the two eligible studies examining tea intervention on QoL, one study used tea beverages, while the other study used a green tea-based lozenge. Participants also differed in being at risk or diagnosed with xerostomia and hyposalivation (Table 2). Abdul-Wahab et al. [38] conducted a single-blind, parallel RCT involving 40 adults (age 22–33 years; 92.5% female) diagnosed with gingivitis. Participants were assigned (20/group) to drink either green tea or matcha tea twice daily. The tea preparation method was not reported. Participants maintained their usual oral hygiene practices and, at the end of the one-month intervention, completed a self-administered 14-item Oral Health Impact Profile (OHIP-14) questionnaire assessing oral health as it relates to QoL. Responses were recorded using a 4-point Likert scale, with lower scores indicating better QoL.

The results suggested that tea consumption improved overall QoL. There were no significant differences in scores between the green tea (1.17 ± 2.01) and matcha tea (0.74 ± 1.41) groups. However, the green tea group reported lower physical pain scores (*p* = 0.02), and the matcha tea group reported lower psychological discomfort scores (*p* = 0.046). The authors suggested that matcha tea reduced stress due to its higher theanine content altering neurotransmitters and higher arginine content increasing vasodilation. However, chemical analysis of the teas did not include amino acids. Alternatively, the observed differences may have been due to green tea’s anti-inflammatory catechins reducing localized pain, while matcha tea’s higher catechin content exerts systemic effects. The increased severity of periodontal disease has been associated with lower SFR and salivary pH [54]; however, gingivitis is an early stage of periodontitis.

In patients with Sjögren’s syndrome, xerostomia is a major contributor to reduced QoL [59]. A phase II double-blind, placebo-controlled RCT [39] included 60 Sjögren’s syndrome patients (age 21–74 years; 96% female) with hyposalivation and xerostomia. The participants were randomly assigned (n = 30/group) to receive lozenges containing a patented formulation or a placebo without green tea extract. Participants were instructed to take their assigned lozenge once every 4 h (no more than 6 times a day) for 8 weeks. A QoL questionnaire was used to measure the severity of xerostomia complaints. After fasting for 1.5 h, USFR was measured by the draining method, followed 5 min later by chewing wax to collect SSFR at baseline and at 1, 4, and 8 weeks.

At the end of the 8-week intervention, the green tea extract intervention increased USFR by 3.8-fold and SSFR by 2.1-fold compared to baseline, with no significant changes in the placebo group. Both groups showed significantly improved QoL, with no significant difference between the groups. However, the placebo lozenge was not inert but contained 500 mg of xylitol. A systematic review reported that xylitol increases saliva flow and pH [60]. These findings suggest that subjective symptoms of dry mouth can improve even without measurable changes in saliva output.

Regarding participants’ oral health status, both gingivitis and Sjögren’s syndrome participants showed improvements in QoL following tea-based interventions; however, studies were limited and heterogeneous. Both RCTs were rated as having a moderate risk of bias due to uncertainty regarding adherence to the intervention (Figure 3A). Additionally, the limited number of studies and high heterogeneity in tea delivery form (i.e., beverage versus lozenge), which may influence the oral exposure and bioavailability of tea bioactive compounds, limited comparability across studies. Consequently, no definitive conclusions could be made about the effectiveness of tea-based interventions on QoL. More studies that include both QoL and saliva biomarkers, as well as a wider range of tea types, are needed.

### 4.5. Strengths and Limitations

The strength of this narrative review was its use of a systematic approach to increase transparency and reproducibility. Another strength of this review was the inclusion of healthy and at-risk patients and patients with dry mouth, which improves the generalizability of the findings, given that xerostomia affects ~1 in 5 people in the general global population [61]. Across studies, 67% of participants were between the ages of 17 and 39 years, a population that is often underrepresented in xerostomia research. Young adults are also an increasingly relevant at-risk group due to a significant rise in medication usage linked to early-onset chronic diseases and mental health conditions [62,63]. Evidence indicates that xerostomia is more strongly associated with the use of medication and underlying disease than with age [64].

**Figure 4:**
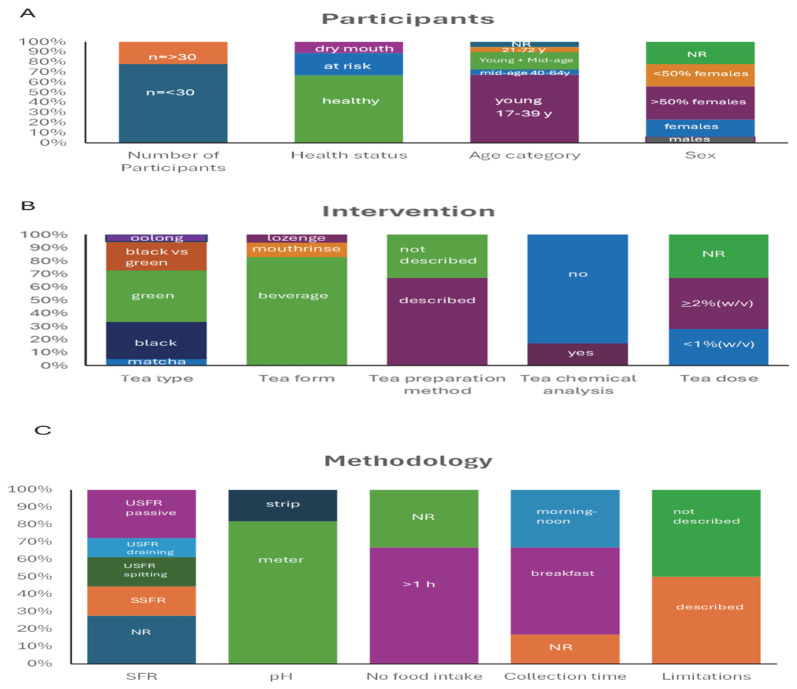
Study characteristics of (**A**) participants, (**B**) interventions, and (**C**) methodological features. Abbreviation: NR, not reported; SSFR, stimulated salivary flow rate; USFR, unstimulated salivary flow rate.

The reporting of limitations is essential for the accurate interpretation of clinical trial findings. Stockli et al. [65] found the reporting of study limitations in dental manuscripts to be suboptimal. In the current review, 50% of the eligible studies reported limitations, with a small sample size being the most frequently cited constraint. Of the eligible studies, 78% had <30 participants. Figure 4A–C shows the frequency of study characteristics. Despite sex differences in SFR [66], 22% of the studies did not indicate participant sex distribution.

A major limitation across the eligible studies was the substantial methodological and clinical heterogeneity, which complicated direct comparisons between studies and the interpretation of tea-specific effects. Although the inclusion of participants with diverse health statuses improved generalizability, differences in health status can influence responsiveness to tea interventions. Healthy individuals comprised the majority of the participants (67%), which potentially limited the observable improvement, as they generally have normal salivary gland function and higher baseline SFR than participants with salivary dysfunction, which reduces the potential for measurable improvement. Consequently, findings observed in healthy cohorts may be more reflective of preventative benefits rather than therapeutic efficacy. This highlights the need for greater inclusion of at-risk patients and those diagnosed with hyposalivation and xerostomia.

Other limitations were the interventions. The studies included a wide range (1.33–15%) of tea doses, with no rationale provided for the selected doses. Post-intervention measurements varied from immediately to at 30 min, which can influence the bioavailability and oral exposure time of bioactive compounds such as catechins and polyphenols, thereby affecting salivary stimulation and pH responses. Additionally, 33% of the studies did not describe the tea preparation method. Heterogeneity in steeping temperature and time and with regard to loose tea versus tea bags can influence tea chemical composition and intrinsic tea pH. However, only 17% of the studies performed chemical analysis of the tea prior to the intervention [22,23,38].

Another key limitation of the studies was heterogeneity in the measurement of outcomes (Figure 4C). The method used to collect saliva was not reported in 28% of the studies. Measurement of USFR was reported in 55% of the studies, 17% reported SSFR, and one study reported both [39]. USFR and SSFR involve different glands, saliva composition, and thresholds for diagnosing hyposalivation [47,67]; these differences can confound comparison across studies. Furthermore, the studies measuring USFR used different collection methods (i.e., drooling, draining, spitting, or aspiration), which produce different levels of mechanical stimulation that influence salivation. Even minor differences in saliva collection techniques have the potential to lead to variable results, such as the time of day and duration of abstention from eating, drinking, and brushing teeth [67]. In this review, 33% of the studies did not report whether participants abstained from these activities or specify the time of day when samples were collected.

## 5. Summary and Conclusions

Overall, the studies suggest that tea consumption, particularly green and black tea, influences salivary parameters; however, results vary depending on the tea preparation methods, outcomes measured, and population studied. Most participants were healthy young adults and predominantly female. Women represented 64% of study participants, with three studies exclusively conducted among women [26,32,34]. This is relevant given the higher prevalence of xerostomia in females [4]. Tea stimulated salivary secretion in at-risk patients and those with reduced salivary flow [25,26,31,36,37,38,39], with less consistent results in healthy populations. Accordingly, clinical relevance should be interpreted primarily from studies involving at-risk and xerostomia populations, whereas findings in healthy individuals with normal baseline salivary function likely reflect preventive or mechanistic effects rather than therapeutic efficacy.

All the tea types transiently increased pH above baseline level, with green tea generally showing the strongest alkalizing effects [25,28,31,33,40,49,50]. Green tea-based interventions improved oral-health-related QoL; however, the evidence was limited to two studies [38,39].

Despite generally positive findings, definitive conclusions were limited by substantial heterogeneity in study designs, participant characteristics, and methodologies. Overall, the studies were evenly rated as having either a high or moderate risk of bias (Figure 3A,B). This review’s critical evaluation of studies highlights the need for additional research to support evidence-based recommendations. Future research should prioritize well-designed RCTs with larger sample sizes. Among the RCTs included in this review, the main source of bias was due to a lack of blinding, unclear randomization methods, and unclear baseline comparability. Using standardized saliva collection protocols such as those proposed by Navazesh et al. [47] can improve comparability and reproducibility across studies. In addition, chemical analysis of tea before intervention can provide mechanistic insights, while more representative dosing is needed, as 58% of studies used concentrations above 2% w/v, exceeding typical consumption. Studies should also include inert controls (e.g., water). Finally, adopting an integrative approach that includes both objective (SFR, pH) and subjective (QoL) outcomes can contribute to a better understanding of the relationship between physiological changes and symptom relief.

As a widely consumed, accessible, and low-cost beverage, tea can serve as a simple dietary modification to support oral health. Oral dryness is a complex condition of physiological impairment of salivary function with or without perceived symptoms. Because effective management depends on the accurate diagnosis and identification of underlying conditions, interventions that alleviate symptoms while improving SFR and salivary pH without systemic side effects may be particularly valuable in supporting both oral health and overall well-being.

## Figures and Tables

**Table 1:** Participants, Intervention, Comparator, Outcomes, Study design (PICOS) framework.

Participants	Inclusion	Exclusion
Population	Age ≥ 17 years Healthy individuals At-risk individuals Individuals with hyposalivation and/or xerostomia	Age < 17 years (due to potential caffeine sensitivity and need for supervision)
Intervention	Tea derived from *Camellia sinensis* including: Black tea Green tea Oolong tea Matcha tea Delivered as: Beverage Lozenge Mouth rinse	Herbal teas or infusions not derived from *Camellia sinensis* Tea type not reported Purified tea extracts (e.g., catechins, EGCG)
Comparator	Other beverages not made from *Camellia sinensis* Other mouth rinses not made from *Camellia sinensis* Placebo controls No intervention (pre- or post-study design)	
Outcomes	At least one of: Salivary flow rate (SFR) Salivary pH Quality of life (QoL)	Studies not measuring at least one of the specified outcomes
Study Design	Human intervention trials	*In vitro* studies *In vivo*, animal, pre-clinical studies Observational studies (Cross-sectional, cohort, case-control) Case reports or case series

**Table 2:** Summary of eligible studies.

Reference	Participants	Intervention	Comparator	Duration	Results
Salivary Flow Rate (mL/min)					
Chong et al. (2023) [22]	Healthy (age 22–31 yrs) n = 12 (6F, 6M)	Black tea Oolong tea 3.33% (w/v)	None Pre- and post-intervention	Baseline, 3, 30 min	USFR (NS) Oolong tea (0.35–0.37) Black tea (0.32–0.37)
Chong et al. (2021) [23]	Healthy (age 22–31 yrs) n = 12 (6F, 6M)	Black tea 3.33% (w/v)	None Pre- and post-intervention	Baseline, 3, 30 min	USFR (NS) 0.08–0.54
Meng et al. (2024) [24]	Healthy (age 20–30 yrs) n = 20/group (13F, 7M)	Hot/cold black tea 3.33% (w/v)	Hot/cold water Pre- and post-intervention	Baseline, 3, 30 min	USFR (NS) Tea ~0.35–0.41
Fitri et al. (2024) [25]	At risk (caries) (age 18–25 yrs) n = 13/group (24F, 2M)	Black tea 1.33% (w/v)	None Pre- and post-intervention	30 min	↑ USFR Caries-free (0.56–0.89) Caries (0.52–0.74) NS caries vs. caries-free
Tampubolon et al. (2020) [26]	Xerostomia (age 45–65 yrs) n = 20/group (20F)	Green tea 15% (w/v)	Water Pre- and post-intervention	3 min	↑ USFR Green tea (0.15–0.43)
Salivary pH					
Demir et al. (2017) [27]	Healthy (age 18–23 yrs) n = 28 (17F, 11M)	Green tea Dose (ND)	Yogurt drink	Baseline, 1, 10, 30 min	↑ Green tea vs. yogurt drink 1 and 30 min 7.04–7.42
Shirzaiy et al. (2021) [28]	Healthy (age 20–22 yrs) n = 50 (50M)	Black tea 1.33% (w/v) Green tea 2% (w/v)	None Pre-and post-intervention	Baseline, immediately, 5, 10 min	Both teas increased pH 7.15~7.51 ↑ Green vs. black tea
Awadalla et al. (2011) [29]	Healthy (age 21–46 yrs) n = 24 (13M,12F)	Green tea Black tea 2%(w/v)	None Pre- and post-intervention	7 min	↓ 6.92–6.43
Balappanavar et al. (2013) [30]	Healthy (age 18–25 yrs) n = 10/group (sex F5, M5)	0.5% (v/v) green tea	0.2% chlorhexidine	Baseline, immediately, 1, 2, 3 wks	↑ Green tea at 3 wks 4.94–6.44 ↑ Green tea vs. chlorhexidine
Srinidhi et al. (2014) [31]	At risk (caries) (age 18–20 yrs) 10/group (sex NR)	Black tea Green tea 1 tea bag 90 mL water	None Pre- and post-intervention	Baseline, immediately 5, 10 min	↑ Green tea Caries-free (7.00–8.55) Caries (6.97–8.73) ↑ Black tea Caries-free (6.98–8.73) Caries (6.89–8.79) ↑ pH green tea vs. black
Salivary Flow Rate (mL/min) + pH					
Mardiati & Wiradona (2023) [32]	Healthy (age NR) n = 20 (F)	Black tea Dose (NR)	None Pre- and post-	NR	↑ USFR 0.85–1.90 ↑ pH 6.9–7.2
Shalal (2017) [33]	Healthy (age 25–30 yrs) n = 255 sex (NR)	Black tea 50% (w/v)	None Pre- and post-	3 min	↑ USFR 0.53–0.56 ↑ pH 6.04–6.13
Astrina et al. (2023) [34]	Healthy (age 17–25 yrs) n = 18 (F) females	Green tea 0.25% (w/v)	None Pre- and post-	5 min	↑ USFR 2.17–3.33 ↑ pH 6.87–7.28
Shetty et al. (2020) [35]	Healthy (age 18–23 yrs) n = 30/group (24M, 36F)	Green tea Black tea 2% (w/v)	None Pre- and post-	Baseline, 3, 6, 9 min	USFR ↑ Green tea (0.39–0.63) * ↑ Black tea (0.37–0.79) * NS black tea vs. green tea pH NS green tea (7.12–7.11) * ↑ Black tea (7.08–7.20) * ↑ Black tea vs. green tea
Masoumi et al. (2016) [36]	Healthy + periodontitis (age 20–50) n = 10/group sex NR	Green tea Black tea 1 bag in 250 mL	None Pre- and post-	Baseline, 1, 5, 10 min	USFR Green tea NS healthy (0.83–0.85) ↑ Periodontitis (0.64–0.69) Black tea NS healthy (0.96–0.96) ↑ Periodontitis (0.88–0.87) pH Green tea NS healthy (6.84–6.86) ↑ Periodontitis (6.84-6.91) Black tea NS healthy (6.81–6.82) NS periodontitis (6.94–6.97) ↑ Green vs. black USFR + pH
Gad (2024) [37]	At risk (caries) age NR 12/group sex NR	Green tea Matcha tea Dose NR	Pre- and post-	Baseline, immediately 5, 10 min	USFR Immediately, 5 min ↑ Green tea (0.92–1.58) ↑ Matcha tea (0.71–0.94) ↑ Green tea vs. matcha tea pH All time intervals ↑ Green tea (7.24–7.57) ↑ Matcha tea (7.21–7.42) ↑ Green vs. matcha tea
QoL					
Abdul-Wahab et al. (2025) [38]	At risk (gingivitis) (age 27–31 yrs) n = 20/group (3M, 37F)	Matcha tea Green tea Dose NR	Pre- and post-	1 month	↑ QoL matcha ↑ QoL green tea
De Rossi et al. (2014) [39]	Xerostomia age 21–74 yrs) n = 30/group (2M, 58 F)	Green tea lozenges 6/day Dose NR	500 mg xylitol lozenge	8 weeks	Lozenge vs. placebo ↑ USFR (3.8 fold) ↑ SSFR (2.1 fold) ↑ QoL NS lozenge vs. placebo

Abbreviations: M, males; F, females; NR, not reported; yrs, years, QoL, quality of life, ↑ increased, ↓ decreased, * time not provided.

## Data Availability

No new data were created or analyzed in this study. Data sharing is not applicable to this article.

## Supplementary Materials

The following supporting information can be downloaded at: https://www.mdpi.com/article/10.3390/dj14060363/s1, Table S1: Synthesis-Without-Meta-analysis-(SWiM)-reporting-items.

## Abbreviations

The following abbreviations are used in this manuscript:

AQP5	Aquaporin-5
EGCG	Epigallocatechin-3-gallate
OHIP-14	Oral Health Impact Profile-14
PICOS	Participants, Intervention, Comparator, Outcomes, Study design
PRISMA	Preferred Reporting Items for Systematic Reviews and Meta-Analyses
QoL	Quality of life
RCT	Randomized controlled trial
SARS-CoV-2	Severe Acute Respiratory Syndrome Coronavirus 2
SFR	Salivary flow rate
SSFR	Stimulated salivary flow rate (used once in QoL section)
USFR	Unstimulated salivary flow rate

## Appendix A

**Table A1:** Search Strategy.

Database	Search Words	# of Articles
PUBMED 1982 to 2025	(“green tea”[All Fields] OR “black tea”[All Fields] OR “oolong tea”[All Fields] OR “matcha tea”[All Fields] OR “Camellia sinensis”[All Fields] OR “polyphenol”[All Fields] OR “catechin*”[All Fields]) AND (“dry mouth”[All Fields] OR “xerostomia”[All Fields] OR “saliva*”[All Fields] OR “Sjogren”[All Fields] OR “head and neck radiation”[All Fields])	455
SCOPUS 1965 to 2025	(“green tea” OR “black tea” OR “oolong tea” OR “matcha tea” OR camellia* OR polyphenol* OR catechin* ) AND ( “dry mouth” OR xerostomia OR hyposalivation OR saliva* OR sjogren OR “head neck radiation”)	1044
WEB OF SCIENCE 1900–2025	(“green tea” OR “black tea” OR “oolong tea” OR “matcha tea” OR camellia* OR polyphenol* OR catechin* ) AND ( “dry mouth” OR xerostomia OR hyposalivation OR saliva* OR “Sjögren’s” OR “head neck radiation”)	1009
COCHRANE LIBRARY 1980–2025	In title and abstract in clinical trials (green tea OR black tea OR oolong tea OR matcha tea OR camellia OR polyphenol OR catechin) AND (dry mouth OR xerostomia OR hyposalivation OR saliva* OR Sjogren OR radiation)	246
Google Scholar, Journal of Dentistry	Hand Search	7
Total		2754

## Appendix B

**Table A2:** Screening Form.

Q1. Is the article an original study?	Yes No If “no”, submit form without proceeding further. If unsure, submit to full text review	Exclude: Review articles, systematic reviews, scoping reviews, narrative reviews, systematic reviews and meta analysis
Q2. Is the study peer-reviewed?	Yes No If “no”, submit form without proceeding further. If unsure, submit to full text review.	Exclude: Study protocols, conference paper/abstracts, thesis, gray literature.
Q3. Does the article involve human subjects?	Yes No If “no”, submit form without proceeding further. If unsure, submit to full text review.	Exclude: Animals studies, *in vitro* studies
Q4. Is the article a human intervention trial?	Yes No If “no”, submit form without proceeding further. If unsure, submit to full text review.	Exclude: observational studies
Q5. Are the subject’s adults (age ≥ 17 years)?	Yes No If “no”, submit form without proceeding further. If unsure, submit to full text review.	Exclude: children and adolescents
Q6. Does study include tea from *Camellia sinensis*?	Yes No If “no”, submit form without proceeding further. If unsure, submit to full text review.	Exclude: herbal tea or infusions, purified tea extracts (e.g., catechins, EGCG)
Q7. Does the study include at least one outcome of interest. Salivary flow rate, salivary pH, and/or Quality of life	Yes No If “no”, submit form without proceeding further. If unsure, submit to full text review.	
Q8. Is an original peer-reviewed article?	Yes No If “no”, submit form without proceeding further. If unsure, submit to full text review.	Exclude: systematic literature reviews, narrative reviews, thesis, conference abstracts, grey literature
Q9. Is article written in English?	Yes No If “no”, submit form without proceeding further. If unsure, submit to full text review.	

## Appendix C

**Table A3:** Data Extraction Form.

Question	Options	Comments
Q1. What is the title of the paper?	Specify	
Q2. What country was the study conducted?	○ United States ○ UK ○ Canada ○ Australia ○ Other	If other, specify
Q3. Was the study randomized?	○ Yes ○ No	
Q4. Was the study blinded?	○ Yes ○ No	
Q5. What was the tea type?	○ Green ○ Black ○ Oolong ○ Matcha	
Q6. What was the mode of delivery of the intervention?	○ Beverage ○ Mouth rinse ○ Other, Specify ○ Unclear	
Q7. What was the intervention dose?	Specify	
Q8. How was tea prepared	Specify	
Q9. What was there a chemical analysis of the tea prior to initiation of the intervention?	○ Yes ○ No	
Q10. What was the comparator?	○ Placebo, control ○ Without tea ○ None (pre- and post-intervention)	
Q11. Where participants’ fasted prior to collection of saliva?	○ No ○ Yes, if yes how long? ○ Not reported	
Q12. What was the study duration?	Specify	
Q13. What is the total number of participants & number of participants per group?	Specify	
Q14. What was the age of the participants?	○ Placebo, control ○ Without tea ○ None (pre & post)	
Q15. What was the sex of the participants?	○ Male only ○ Female only ○ ≥50% female ○ <50% female	
Q16. What heath status are participants at risk for?	Specify	
Q17. What is the total number of participants & number of participants per group?	Specify	
Q18. What outcomes were measured?	○ salivary flow rate ○ salivary pH ○ quality of life	
Q19. Were participants fasted prior to collection of saliva?	○ No ○ Yes, if yes how long? ○ Not reported	
Q20. Time of day the saliva samples were collected	Specify	
Q21. How was salivary pH measured?	○ pH meter ○ pH strip ○ Not reported	
Q22. How was salivary flow measured?	○ USFR passive drooling ○ USFR draining ○ USFR aspiration ○ SSFR (chewing wax) ○ Not reported	
Q23. Were limitations reported	○ Yes ○ No If yes, specify	
Q24. What is the study’s funding sources?	Specify	
